# Editorial: Interplay between environmental drivers and genetic or epigenetic predispositions on gastrointestinal cancer evolution

**DOI:** 10.3389/fonc.2025.1754708

**Published:** 2026-01-14

**Authors:** Xunye Xu, Daoyuan Wang, Xudong Huang, Caihong Wang

**Affiliations:** 1 Laboratory of Surgical Oncology, Department of Gastroenterological Surgery, Peking University People’s Hospital, Beijing, China; 2 State Key Laboratory of Oncology in South China and Guangdong Provincial Clinical Research Center for Cancer, Sun Yat-sen University Cancer Center, Guangzhou, China

**Keywords:** environmental determinants, epigenetic, gastrointestinal cancer (gi cancer), genetic, multilayered networks

Cancer is clearly a genetic disease, and previous studies have mainly focused on the roles of intrinsic genetic drivers and somatic mutations in oncogenes and tumor suppressor genes in driving tumor development (1). However, accumulating evidence indicates that non-mutagenic promotion can also mediate clonal selection for malignant phenotypes. The evolution of gastrointestinal (GI) cancer is shaped by the interplay of genetic susceptibility, epigenetic modifications, and extracellular environmental factors, which collectively drive a highly heterogeneous and dynamically adaptive tumor trajectory. These multi-level and comprehensive research perspectives are changing the way we approach the prevention and treatment of gastrointestinal tumors (2, 3).

Recent studies in this research Topic have provided key evidence, from the global disease burden increase caused by high BMI, to the carcinogenic effects of epigenetic genes such as SMYD2 and BTBD19, to the adaptation of the tumor microenvironment mediated by the hypoxia-TRIM21-ID1 axis, and the remodeling of the tumor microenvironment and promotion of immune escape by the metabolic products of the intestinal microbiota. Collectively, these studies reveal that cancer is a systems-level process driven by the coordinated interaction between genetic, epigenetic, and environmental factors. In gastrointestinal malignancies, this multilayered framework is particularly evident: genetic alterations provide the baseline oncogenic potential, epigenetic regulation governs dynamic shifts in cellular states, and environmental pressures determine the selective forces that shape tumor initiation and progression.

While the genetic basis of cancer lies in driver mutations and clonal selection, mutation-centric models alone cannot adequately explain carcinogenesis or clinical heterogeneity. Epigenetic regulation provides a crucial complementary mechanism that controls gene expression without altering the DNA sequences or structures, thereby reshaping cell fate (4). Wang et al. demonstrated that the histone methyltransferase SMYD2 is significantly upregulated and promotes cell proliferation, migration, and immune evasion via multiple oncogenic pathways (5). SMYD2 methylates and suppresses tumor suppressors such as p53 and RB, while activating PI3K–AKT and STAT3 signaling, thereby enhancing tumor growth and drug resistance. This highlights the role of epigenetic control during the early stages of carcinogenesis. Yang et al. identified BTBD19, a member of the BTB/POZ-domain family, as a promoter of tumor progression through modulation of immune infiltration and inflammation-related pathways. Its overexpression correlates with poor prognosis, suggesting its potential as a prognostic biomarker. In summary, genomic mutations lay the foundation for cancer occurrence, while epigenetic modifications determine how this process is activated or suppressed in different microenvironments. Together, they constitute the “intrinsic driving force system” of gastrointestinal cancer evolution.

The GI tract, constantly exposed to environmental stimuli, is profoundly influenced by metabolic and inflammatory signals (6, 7). Obesity, hypoxia, and metabolic reprogramming not only induce DNA damage but also modulate immune and metabolic pathways that drive malignant progression. Yao et al. analyzed Global Burden of Disease 2021 data (1990–2021) and revealed a steady rise in obesity-associated colorectal, liver, and pancreatic cancers, with an estimated annual percent change exceeding 4% for pancreatic cancer. Although high-SDI regions achieve better overall cancer management, obesity remains a significant risk factor. These findings highlight the global epidemiological impact of metabolic stress on GI cancer and its importance for public health interventions. Cheng et al. further elucidated a hypoxia-mediated mechanism in pancreatic ductal adenocarcinoma (PDAC). Hypoxia inhibits the E3 ubiquitin ligase TRIM21, which stabilizes ID1 (Inhibitor of Differentiation 1), thereby promoting epithelial–mesenchymal transition, invasiveness, and chemoresistance. The hypoxia–TRIM21–ID1 axis represents a pivotal adaptive pathway and a potential therapeutic target in the tumor microenvironment. Clinically, such interactions explain diverse pathophysiological phenomena. For instance, Peng et al. reported cases of synchronous primary liver and lung cancers that may have arisen from a shared genetic predisposition, exposure to environmental carcinogens, or immune dysfunction. Similarly, BTBD19-associated immune infiltration in colorectal cancer exemplifies the bidirectional regulation of gene expression and tumor immunoecology. Guo and Wang provided an in-depth analysis of metabolic reprogramming in Colorectal Cancer (CRC) and linked it to the co-metabolism of the gut microbiota, revealing a complex pathway from pathogenesis to precision therapies. Integrating these findings provides a holistic understanding of GI cancer as a disease influenced by both genetics and the environment.

## Future directions: toward multidimensional precision oncology

The collective evidence underscores that GI tumorigenesis is driven by the interplay of genetic, epigenetic, and environmental determinants.

- Epigenetic regulation: SMYD2 activates multiple oncogenic cascades, and BTBD19 modulates immune and inflammatory pathways to promote tumor growth.
- Environmental influence: Obesity, microenvironmental hypoxia, and gut microbial metabolites can all influence tumor development.
- Integrative susceptibility: The co-occurrence of multiple primary malignancies indicates shared risk determinants.

## Future directions and clinical implications

With advances in multi-omics and AI technologies, integrating genetic, epigenetic, and environmental data now enables a more comprehensive understanding of tumors. However, key challenges remain, and future research should prioritize the following directions:

1. Multi-omics integration: Combine genomic, epigenomic, and metabolomic data to construct individualized risk networks for the precise prediction of disease progression and therapeutic response.
2. Microenvironment Model Construction: Establish GI cancer organoid or microfluidic chip models that incorporate the microbiota to simulate dynamic *in vivo* feedback loops for drug screening and mechanistic validation.
3. Epigenetic pharmacology: Develop small molecules targeting regulators, such as SMYD2, to reprogram gene expression, and explore combination strategies with gene-targeted and immunotherapies.
4. AI-driven prevention: Use artificial intelligence to identify high-risk patterns and enable early screening and lifestyle interventions.
5. Global collaboration and policy: Given the worldwide rise in obesity and metabolic disorders, cross-regional cooperation and public health initiatives are essential.

## Conclusion

GI cancers arise from the coordinated influence of intrinsic and extrinsic pathogenic forces. Intrinsic drivers, including genetic mutations and epigenetic regulators such as SMYD2 and BTBD19, establish the molecular foundation and functional plasticity of malignant evolution. In parallel, extrinsic pressures, such as high BMI–associated metabolic stress, gut microbiota–derived metabolites, hypoxia-induced microenvironmental adaptation, and the complex interplay of environmental exposures reflected in synchronous primary liver and lung cancers, collectively shape the selective landscape that directs tumor progression. Together, these converging insights support a systems-level model in which GI cancer evolution emerges from the dynamic integration of intrinsic molecular programs and extrinsic ecological forces, offering mechanistic targets for precision therapies and informing preventive strategies to mitigate the growing global burden of gastrointestinal malignancies.
